# Impact of Atrial Fibrillation on Inpatient Outcomes Among Hospitalized Patients With Multiple Myeloma

**DOI:** 10.7759/cureus.25252

**Published:** 2022-05-23

**Authors:** Inimfon Jackson, Aniekeme S Etuk, Nsikak Jackson

**Affiliations:** 1 Internal Medicine, Einstein Medical Center Philadelphia, Philadelphia, USA; 2 Internal Medicine, Thomas Hospital Infirmary Health, Fairhope, USA; 3 Management, Policy and Community Health, University of Texas Health Science Center at Houston, Houston, USA

**Keywords:** predictors, factors, hospital-associated outcomes, inpatient outcomes, multiple myeloma, atrial fibrillation

## Abstract

Background

Though multiple myeloma (MM) patients have been reported to have the highest risk of atrial fibrillation compared to other cancer patients, studies are lacking on the impact of atrial fibrillation on health outcomes in this population. In this study, we examined the impact of atrial fibrillation on inpatient outcomes among hospitalized patients with MM.

Methodology

Retrospective cohort analyses were conducted using National Inpatient Sample data from 2016 to 2018. Descriptive analyses were performed to explore the prevalence of atrial fibrillation among MM patients. Multivariable logistic and linear regression models were used to examine the association between atrial fibrillation and inpatient all-cause mortality, length of stay, and total hospital charges among hospitalized patients with MM.

Results

Overall, 13.1% of the patients reported having atrial fibrillation. MM patients with atrial fibrillation had 1.2 times (adjusted odds ratio (AOR) = 1.16; 95% confidence interval (CI) = 1.05-1.29) higher odds of inpatient all-cause mortality when compared to those without atrial fibrillation. They were also 1.3 times (AOR = 1.29; 95% CI = 1.23-1.35) more likely to have a length of stay of more than five days relative to five days or less. Additionally, MM patients with atrial fibrillation had $8,020 (95% CI = $5,495.2-$10,546.3) higher hospital costs when compared to their counterparts without atrial fibrillation. Stratified results by the use of anticoagulation further showed that MM patients who were not using anticoagulation had bad health outcomes, reporting higher odds of inpatient all-cause mortality (AOR = 1.40; 95% CI = 1.25-1.57), a longer length of hospital stay of more than five days (AOR = 1.44; 95% CI = 1.36-1.53), and total hospital charges (β = $14,772.5; 95% CI = $11,467.8-$18,077.3).

Conclusions

Our findings stress the need for monitoring and possible screening to detect atrial fibrillation in MM patients as anticoagulation helps improve mortality in these patients. Medication reconciliation remains a key component of hospital admissions/discharges and may help in decreasing the length of stay and healthcare costs.

## Introduction

Multiple myeloma (MM) is the second most common hematological malignancy associated with a five-year survival rate of approximately 54% [1-3]. It is a plasma cell dyscrasia more common in men and the African American population. Over the years, novel therapies have been developed to improve survival in patients with this malignancy [3-5]. However, MM patients are shown to have multiple comorbidities that worsen their survival rates [6]. A recent study published in the Journal of the American College of Cardiology reported that MM patients had the highest risk of atrial fibrillation when compared to other cancer patients [7]. Atrial fibrillation is a key public health issue and is reported to account for high healthcare costs in developed countries [8]. It is frequently associated with cardiac diseases and comorbidities [8,9].

Patients with MM have a higher risk of concomitant heart failure related to previous therapy or cardiac amyloidosis [10-12]. While atrial fibrillation has been reported to be associated with a higher risk of mortality and healthcare expenditure in the general population and among cancer patients [7,13], there are no studies exploring the impact of atrial fibrillation on hospital-associated outcomes among MM patients. This study aimed to examine the impact of atrial fibrillation on inpatient mortality, length of stay, and total hospital charges among hospitalized patients with MM. Furthermore, the association between atrial fibrillation and these inpatient outcomes was assessed and stratified by the use of anticoagulation.

## Materials and methods

Data source

The Healthcare Cost and Utilization Project National Inpatient Sample (HCUP-NIS) data collected between 2016 and 2018 were utilized for the analyses [14]. It is a database sponsored by the Agency for Healthcare Research and Quality (AHRQ), and the sample covers over 97% of the US population. The survey is conducted every year and includes up to 20% of hospital admissions to community hospitals in the United States. Additional information about the administration, methodology, and data collection is described elsewhere [15]. Because this data is de-identified and publicly available, it does not require institutional review board review based on the code of federal regulations.

Study population

All patients aged 18 years and above with a diagnosis of MM, identified using the following International Classification of Disease, Tenth Revision, Clinical Modification (ICD-10-CM) codes: C90.0 (MM), C90.00 (MM not having achieved remission), C90.01 (MM in remission), and C90.02 (MM in relapse), were selected. In total, 68,279 hospitalizations had a diagnosis of MM, and after excluding patients less than 18 years old (0.02% of hospitalizations), a total number of 68,267 hospitalizations with a diagnosis of MM were included in the data analyses.

Hospital outcomes

The outcomes evaluated in this study included inpatient all-cause mortality, length of stay, and total hospital charges. All-cause mortality was categorized as yes or no for death during hospitalization versus not, length of stay as five days or fewer and more than five days based on the national average of five days for length of hospital stays for any admission in the United States. Total hospital charge was a continuous variable with units in dollars.

Independent variable

The main independent variable in this study was atrial fibrillation. The ICD-10 code I48 was used to identify patients with a diagnosis of atrial fibrillation, and the variable was categorized into atrial fibrillation versus no atrial fibrillation.

Covariates

Based on previous literature, the covariates selected for inclusion in our study were categorized into sociodemographic and clinical. Sociodemographic covariates included age (<70 years, 70 years and above), gender (male, female), race/ethnicity (non-Hispanic White, non-Hispanic Black, Hispanic, and non-Hispanic other), median household income national quartiles (quartiles 1-4), insurance type (Medicaid, Medicare, private, and other), hospital region (Northeast, Midwest, South, and West), and hospital location (rural, urban non-teaching, and urban teaching). Clinical covariates included type of admission (elective and non-elective), anticoagulation use (yes versus no), and Charlson Comorbidity Index (0-4, 5-7, and 8 or more).

Statistical analyses

Appropriate weights were applied to the data to account for the complex survey design and make the data representative of the general population. Categorical variables were analyzed using counts and frequencies, while continuous variables were analyzed using means and standard deviation. Chi-square tests were used to assess for differences between categorical variables. Sociodemographic variables were explored by atrial fibrillation. Multivariable logistic regression was used to examine the association between atrial fibrillation and inpatient all-cause mortality and length of stay, while multiple linear regression was used to assess the association between atrial fibrillation and total hospital charges among hospitalized patients with MM. Further analyses were conducted and stratified by the use of anticoagulation. Stata SE 16.1 (StataCorp, College Station, TX, USA) was used for all analyses, and statistical significance was determined with a two-sided p-value of <0.05 based on the Wald test.

## Results

Out of 68,267 hospitalizations with a diagnosis of MM, 13.1% reported having atrial fibrillation. Overall, 4.82% reported inpatient all-cause mortality, 56.9% had a length of stay of five days or less, and the mean hospital cost was $78,643.6. The majority of those with atrial fibrillation were non-Hispanic white (75.7%) males (60.9%) aged 70 years and above (74.6%). They were mostly on Medicare (84%) and were admitted non-electively (90.1%) in urban teaching hospitals (71%). Among those without a diagnosis of atrial fibrillation, 52% were aged less than 70 years, 54.6% were males, and 61.8% were non-Hispanic whites. A greater proportion of them was also admitted non-electively (83%) in urban teaching hospitals (75.2%) and on Medicare (65.9%) (Table 1).

**Table 1:** Sociodemographic and hospital-level characteristics of multiple myeloma patients hospitalized with and without atrial fibrillation. n = unweighted number of observations; wt% = weighted percentages

	wt%		
	Atrial fibrillation		P-value
	Yes	No	
	n = 8,926	n = 59,341	
Age			<0.001
Less than 70	25.35	52.01	
70 years and above	74.65	47.99	
Gender			<0.001
Male	60.94	54.58	
Female	39.06	45.42	
Race/Ethnicity			<0.001
Non-Hispanic white	75.72	61.81	
Non-Hispanic black	14.29	23.40	
Hispanic	5.29	8.91	
Non-Hispanic other	4.70	5.88	
Median household income national quartiles			<0.001
Quartile 1	22.45	27.42	
Quartile 2	24.17	24.78	
Quartile 3	27.1	24.34	
Quartile 4	26.3	23.46	
Insurance type			<0.001
Medicaid	2.58	6.95	
Medicare	84.00	65.93	
Private	11.56	23.59	
Other	1.86	3.52	
Hospital region			<0.001
Northeast	19.87	21.03	
Midwest	26.25	22.60	
South	35.33	38.46	
West	18.5	17.91	
Hospital location			<0.001
Rural	6.53	6.33	
Urban non-teaching	22.42	18.48	
Urban teaching	71.05	75.19	
Mortality			<0.001
No	93.76	95.40	
Yes	6.24	4.60	
Admission type			<0.001
Elective	9.86	16.95	
Non-elective	90.14	83.05	
Length of stay			<0.001
Five days or less	52.85	57.55	
More than five days	47.15	42.45	
Charlson Comorbidity Index			<0.001
0–4	42.81	62.03	
5–7	42.08	26.45	
⩾8	15.11	11.52	

Adjusted analyses found that MM patients with atrial fibrillation had 1.2 times (adjusted odds ratio (AOR) = 1.16; 95% confidence interval (CI) = 1.05-1.29) higher odds of inpatient all-cause mortality when compared to those without atrial fibrillation. They were also 1.3 times (AOR = 1.29; 95% CI = 1.23-1.35) more likely to have a length of stay of more than five days relative to five days or less. Additionally, MM patients with atrial fibrillation had $8,020 (95% CI = $5,495.2-$10,546.3) higher hospital costs when compared to their counterparts without atrial fibrillation. Further analyses were conducted, stratified by the use of anticoagulation. Among MM patients on anticoagulation, there was no difference in inpatient all-cause mortality (AOR = 0.87; 95% CI = 0.67-1.13) when patients with and without atrial fibrillation were compared. However, MM patients with atrial fibrillation were more likely (AOR = 1.18; 95% CI = 1.07-1.30) to have more than a five-day length of stay compared to five days or less. They also had $3,657.9 higher (95% CI = $445.6-$6,870.2) hospital costs when compared to their counterparts without atrial fibrillation. On the other hand, MM patients with atrial fibrillation who were not on anticoagulation had higher inpatient all-cause mortality (AOR = 1.40; 95% CI = 1.25-1.57), length of stay (AOR = 1.44; 95% CI = 1.36-1.53), and hospital costs (β = $14,772.5; 95% CI = $11,467.8-$18,077.3) relative to those without atrial fibrillation who were not on anticoagulation (Table 2).

**Table 2:** Adjusted analyses of the impact of atrial fibrillation on hospitalization outcomes among patients with multiple myeloma by anticoagulation use. Model adjusted for age, gender, race/ethnicity, hospital region, hospital location, Charlson Comorbidity Index, and insurance type. OR = odds ratio; CI = confidence intervals

		Atrial fibrillation	
	Overall	Anticoagulation use	No anticoagulation use
Mortality	OR (95% CI)		
No	Reference	Reference	Reference
Yes	1.16 (1.05–1.29)	0.87 (0.67–1.13)	1.40 (1.25–1.57)
Length of stay	OR (95% CI)		
Five days or less	Reference	Reference	Reference
More than five days	1.29 (1.23–1.35)	1.18 (1.07–1.30)	1.44 (1.36–1.53)
Total charges	β (95% CI)		
	8,020.8 (5,495.2–10,546.3)	3,657.9 (445.6–6,870.2)	14,772.5 (11,467.8–18,077.3)

## Discussion

Our study highlights the impact of atrial fibrillation on hospitalization outcomes among inpatients with MM. We found that MM patients with a diagnosis of atrial fibrillation had higher odds of inpatient all-cause mortality, length of stay greater than five days, and higher total hospital charges when compared to their counterparts without atrial fibrillation. When stratified by the use of anticoagulation, there was no association between atrial fibrillation and inpatient all-cause mortality but there was an increase in the length of stay and hospital charges. Among those not on anticoagulation, atrial fibrillation led to higher inpatient all-cause mortality, length of stay, and hospital charges. Studies have reported the prevalence of atrial fibrillation among cancer patients ranging between 14% and 20% [16,17]. Similar to other studies conducted among those with cancer, about 13% of hospitalized MM patients had a diagnosis of atrial fibrillation in our study population.

In the general population, atrial fibrillation is known to be associated with a higher risk of mortality [18,19]. Furthermore, atrial fibrillation has been reported to impact mortality among patients with different comorbidities including those with cancer [7]. Compared to other cancer types, MM patients have the highest risk of developing atrial fibrillation, and, to our knowledge, this is the first study to examine the impact of atrial fibrillation on mortality among MM patients [7]. The increased mortality risk of atrial fibrillation is due to its related complications, such as heart failure and embolism, other comorbidities in these patients, and side effects of the treatment strategies, such as bleeding from anticoagulation and anti-arrhythmic drugs [13].

Regardless, anticoagulation is an effective means to reduce mortality and stroke [20]; hence, the need for adherence should be discussed with patients prior to hospital discharge. Hospitalized MM patients may benefit from active screening for atrial fibrillation as this may identify and result in early treatment to prevent complications. Atrial fibrillation has been shown to increase length of stay among patients undergoing procedures [21]. Our study reported increased length of stay among hospitalized MM patients and this may be due to older age and other comorbidities that also need to be managed in these patients. Additional research is needed on ways to decrease the length of stay and minimize the associated hospital charges in this population of patients.

Our study is not without limitations. All-cause inpatient mortality was assessed because cardiovascular disease-specific mortality was not available in the dataset. Bleeding data were not available for patients on anticoagulation. It was also difficult to determine the reasons why some patients were not on anticoagulation, and it is possible that some of them had contraindications to the use of anticoagulation. ICD-10 codes were used to identify patient diagnoses and there is a potential for coding errors. It was impossible to separate atrial fibrillation into valvular or non-valvular as they are all grouped using the same ICD-10 code. Additionally, anticoagulation use could not be further characterized because there is one code for reporting the current use of anticoagulation. Regardless, this study used a large nationally representative database which makes our findings generalizable to the US population. The database did not provide information on the time of initial diagnosis of atrial fibrillation to allow us to establish temporality. However, this study reports novel findings on the inpatient outcomes associated with atrial fibrillation among patients with MM.

## Conclusions

This study examined the impact of atrial fibrillation on hospital-associated outcomes among MM patients. We found that 13% of patients hospitalized with MM reported a diagnosis of atrial fibrillation. Atrial fibrillation was shown to be associated with higher inpatient all-cause mortality, hospital charges, and length of stay among hospitalized MM patients. After stratifying by anticoagulation use, patients who did not use anticoagulation had significantly higher all-cause inpatient morality, longer length of hospital stays, and total hospital charges. Research is needed to explore factors leading to a higher length of stay and hospital costs and ways to decrease these in hospitalized MM patients. While patients need to be educated on the need for medication adherence, there is a need to pay specific attention to medication reconciliation during admission and prior to hospital discharge as this may help improve the length of stay and overall costs of hospitalization.
